# The complete chloroplast genome of *Zanthoxylum piasezkii* Maxim. (Rutaceae) and its phylogenetic analysis

**DOI:** 10.1080/23802359.2020.1866458

**Published:** 2021-02-05

**Authors:** Chong Sun, Xia Liu, Peng Dong, Mi Kuang, Zexiong Chen

**Affiliations:** a College of Landscape Architecture and Life Science, Chongqing University of Arts and Sciences, Chongqing, China; b The Agricultural Technology Extension Station of Chongqing, Chongqing, China

**Keywords:** *Zanthoxylum piasezkii*, complete chloroplast genomes, phylogenetic analysis

## Abstract

*Zanthoxylum piasezkii* Maxim. is a widely distributed species of edible medicinal plant in China. It has been used for traditional spicy condiment and medicinal ingredients for quite a long time. In this study, the complete chloroplast genome sequence of *Z. piasezkii* was first reported and characterized from sequencing data. The complete chloroplast genome was determined to be 158,728 bp in length, consisting of a large single-copy (LSC) region (85,918 bp) and a small single copy (SSC) region (17,612 bp), which were separated by a pair of 27,599 bp inverted repeat (IR) regions. The chloroplast genome is predicted to contain 132 genes, including 87 protein-coding genes, 37 tRNA genes, and 8 rRNA genes. The overall GC content of cpDNA is 38.4%. The phylogenetic analysis of 12 complete chloroplast genomes reveals that *Z. piasezkii* is mostly related to the congeneric *Z. bungeanum*.

*Zanthoxylum piasezkii* Maxim., belongs to the genus *Zanthoxylum* L. (prickly ash) in the Rutaceae family, is an edible medicinal plant and mainly distributed in Sichuan, Shaanxi, Gansu and other provinces in China, growing in dry river valleys or roadside (Zhang et al. 2001). The fruit is rich in volatile oil, which is used as medicine by the people for a long history and has the effect of dispelling cold and strengthening stomach (Yang et al. 2008). However, there is still no complete cp genome was characterized for *Z. piasezkii*. Here, we first characterized the complete cp genome sequence of *Z. piasezkii* based on Illumina pair-end sequencing data, which will provide the basis for the future genetic studies and resource utilization.

Fresh leaves of *Z. piasezkii* were sampled from a young tree grown in Aba Tibetan and Qiang Autonomous Prefecture, Sichuan, China (N31°79′, E103°54′). Fresh leaves were silica-dried and taken back to the laboratory were used for DNA extraction. The voucher specimen (accession number CSHJ2020518) was preserved at the Herbarium College of Landscape Architecture and Life Science, Chongqing University of Arts and Sciences, Chongqing, China and extracted DNA using a modified CTAB method (Doyle and Doyle 1987). The DNA library were sequenced by Hefei Bio&Data Biotechnologies Inc. (Hefei, China) on the BGISEQ-500 platform with PE150 read lengths. The clean reads were assembled the cp genome data using the program NOVOplasty (Dierckxsens et al. 2017). The annotation of the whole genome was performed by CpGAVAS (Liu et al. 2012).

The circular genome of *Z. piasezkii* with a length of 158,728 bp and contained two inverted repeat (IRa and IRb) regions of 27,599 bp, which were separated by a large single copy (LSC) region of 85,918 bp, and a small single copy (SSC) region of 17,612 bp. The cpDNA contains 132 genes, including 87 protein-coding genes, 37 tRNAs gene, and 8 rRNA genes. Among the genes, the eight protein-coding genes, seven tRNA genes, and four rRNA genes were duplicated in IR regions. A total of 19 genes contained two exons and four genes (ycf3, clpP, and two rps12) contained three exons. The overall GC content of *Z. piasezkii* plastome is 38.4%, and the corresponding values in LSC, SSC and IR regions are 36.8%, 33.5%, and 42.5%, respectively. The complete chloroplast genome sequence of *Z. piasezkii* was deposited in GenBank of NCBI (https://www.ncbi.nlm.nih.gov/) database with the accession number MW206785 and Genome Warehouse database with the accession number GWHAOST01000000 (https://bigd.big.ac.cn/gwh/Assembly/10391/show). The raw sequencing data is deposited in the SRA database with the accession number SRX9591236.

To investigate the taxonomic location of *Z. piasezkii*, the phylogenetic analysis was conducted based on 12 species whole cp genome sequences to construct the phylogenetic tree. The 12 complete chloroplast genome sequences were aligned with MAFFT (Katoh and Standley 2013). The maximum likelihood (ML) tree was performed with RAxML version 8 program (Alexandros 2014) using 1000 bootstrap. The results showed that *Z. piasezkii* is closely related to *Z. bungeanum* with 100% bootstrap support values (Figure 1). The whole cp genome sequences of *Z. piasezkii* will pave the foundation for future research to understand the chloroplasts genomic information of the genus *Zanthoxylum* and conservation genetics.

**Figure 1.**
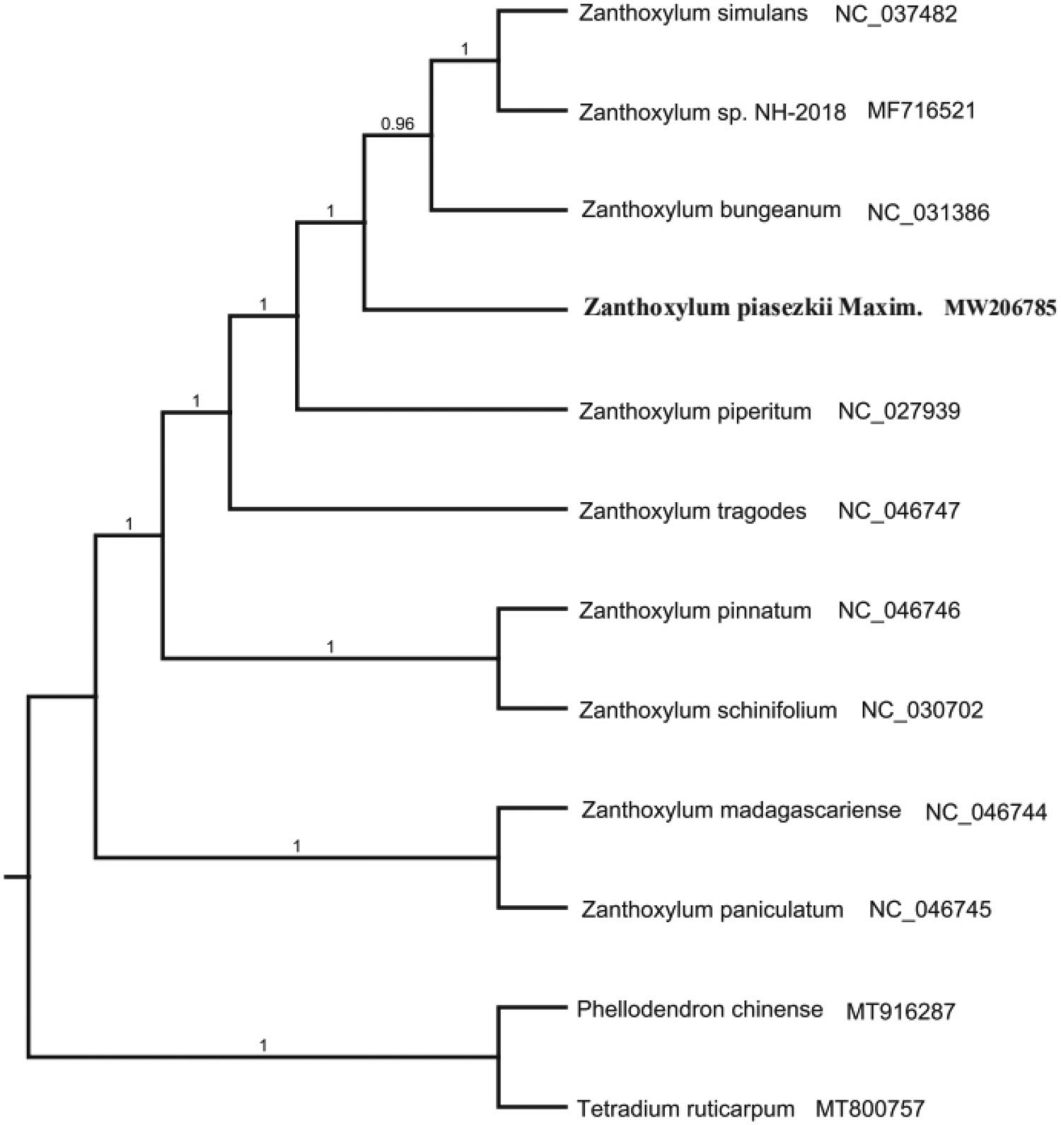
Maximum-likelihood phylogenetic tree of *Zanthoxylum piasezkii* and other related species based on the complete chloroplast genome sequence. The number on each node indicates bootstrap support value.

## Data Availability

The genome sequence data that support the findings of this study are openly available in GenBank of NCBI at https://www.ncbi.nlm.nih.gov/ under the accession number MW206785 and in Genome Warehouse database (https://bigd.big.ac.cn/gwh/) with the accession number GWHAOST01000000 (https://bigd.big.ac.cn/gwh/Assembly/10391/show). The raw sequencing data is deposited in the SRA database with the accession number SRX9591236 (https://www.ncbi.nlm.nih.gov/sra/?term=SRX9591236).
